# The Role of Impulse Oscillometry in Evaluating Disease Severity and Predicting the Airway Reversibility in Patients With Bronchiectasis

**DOI:** 10.3389/fmed.2022.796809

**Published:** 2022-02-25

**Authors:** Cuiyan Tan, Donghai Ma, Kongqiu Wang, Changli Tu, Meizhu Chen, Xiaobin Zheng, Yingjian Liang, Yiying Huang, Zhenguo Wang, Jian Wu, Jin Huang, Jing Liu

**Affiliations:** ^1^Department of Pulmonary and Critical Care Medicine, The Fifth Affiliated Hospital of Sun Yat-sen University, Zhuhai, China; ^2^Guangdong Provincial Key Laboratory of Biomedical Imaging and Guangdong Provincial Engineering Research Center of Molecular Imaging, The Fifth Affiliated Hospital of Sun Yat-sen University, Zhuhai, China

**Keywords:** bronchiectasis, impulse oscillometry, spirometry, plethysmography, airway reversibility

## Abstract

**Background:**

Impulse oscillometry (IOS) can be used to evaluate airway impedance in patients with obstructive airway diseases. Previous studies have demonstrated that IOS parameters differ between patients with bronchiectasis and healthy controls. This study aims to explore the usefulness of IOS in assessing disease severity and airway reversibility in patients with bronchiectasis.

**Method:**

Seventy-four patients with non-cystic fibrosis bronchiectasis who visited our Respiratory Medicine outpatient clinic were consecutively recruited. Spirometry, plethysmography and IOS tests were performed. Patients were stratified into mild, moderate and severe disease according to Reiff, Bhalla, BSI, FACED, and BRICS scores. Airway reversibility was measured by bronchodilation test (BDT) and the result was classified as positive or negative. ROC curves of IOS parameters were used to assess the usefulness of IOS parameters in predicting airway reversibility. Correlations between the IOS, spirometric lung function and bronchiectasis severity parameters were analyzed.

**Results:**

Many IOS parameters, such as airway resistance at 5 Hz (R5), small airways resistance (R5–R20), total airway reactance (X5), resonance frequency (Fres), total airway impedance at 5 Hz (Z5), and peripheral resistance (Rp) increased in patients with bronchiectasis who presented a moderate to severe severity as categorized by the FACED, BSI and Reiff scores. Large airway resistance (R20) and central resistance (Rc) were not significantly different among groups with different bronchiectasis severity. The difference between R5 and R20 (R5-R20) showed 81.0% sensitivity, and 69.8%specificity in predicting the airway reversibility in bronchiectasis with AUC of 0.794 (95%CI, 0.672–0.915).

**Conclusion:**

IOS measurements are useful indicators of bronchiectasis severity and may be useful for predicting the airway reversibility.

## Introduction

Impulse oscillometry (IOS) is a non-invasive and relatively simple method that can be used to detect airway abnormalities. IOS measures airway resistance and reactance using a variant of the forced oscillation technique, and reflect the mechanical properties of both the small and large airways, thus providing information about sites where the obstruction occurs (peripheral or central), severity and respiratory dynamics characteristics. The performance of IOS consists of exerting external pressure signals at different frequencies on the normal tidal breathing and requires minimal patient cooperation. The main measurements of IOS include Respiratory resistance (Rrs), Respiratory reactance (Xrs), Resipratory impedance (Zrs), and the Resonant frequency (Fres) (1).

Bronchiectasis is a chronic airway disease where there is abnormal enlargement of some airways in the lungs. This is normally diagnosed by identification of typical radiographic features in high resolution computed tomography (HRCT) scans, such as bronchial enlargement and wall thickening. These changes are consequences of a complex interaction between various factors, including genetic predisposition, airway dysfunction, inflammatory response, structural diseases and chronic infections (2). Multiple scoring systems have been developed to evaluate the severity of bronchiectasis. The most often used are the modified Reiff (3), FACED (4) and BSI (5) scores. The modified Reiff score is completely based on radiological changes such as bronchial enlargement and number of lobes with pathology, while the FACED and BSI scores integrate radiological and clinical variables, and have been validated to predict the risk of exacerbation, hospital admission and mortality in patients with bronchiectasis.

The applications of IOS in evaluation of lung functions, especially in patients with obstructive lung diseases, are emerging due to its ease of use. IOS measurements suggest that asthma patients present with increased airway resistance and reactance. Preforming IOS in asthma patients is useful for the detection of small airway dysfunction and monitoring asthma control (6, 7). However, the usefulness of IOS in bronchiectasis remain largely unexplored. To our knowledge, there are limited studies that have analyzed the correlation between IOS parameters and bronchiectasis severity. An early study demonstrated that IOS can be used to differentiate bronchiectasis patients from healthy controls (8). Recently, Yamamoto et al. (9), showed that certain IOS parameters correlated with disease severity of bronchiectasis.

This study aims to assess the usefulness of IOS parameters in evaluating the severity of bronchiectasis by analyzing the correlation between the lung function parameters, IOS parameters, and bronchiectasis severity score. Finally, we also aimed to estimate the role of IOS in predicting airway reversibility in non-cystic fibrosis bronchiectasis patients.

## Materials and Methods

### Patient Recruitment

This is a prospective, unicenter study performed in the Fifth Affiliated Hospital of Sun Yat-Sen University (Zhuhai, China), in which patients with non-cystic fibrosis bronchiectasis who visited the outpatient clinic of Respiratory Medicine in June 2017 to May 2019 were consecutively and prospectively recruited into this study. The healthy control group included staff members, interns, graduate students, and people without respiratory symptoms who underwent health check-ups and had a normal chest CT scan.

The diagnosis of bronchiectasis was based on the presentation of typical clinical symptoms (chronic cough, purulent sputum and repeated haemoptysis), signs of localized or persistent moist rales on pulmonary auscultation, and one of the following findings on HRCT: (1) bronchial wall thickening and an enlarged lumen, where the ratio of the bronchus diameter to that of the accompanying artery is 1:1; (2) irregular cystic dilation with a gas-fluid level.

The exclusion criteria were as follows: (1) acute exacerbation within the past 4 weeks, defined as increased amount of sputum, aggravated cough or shortness of breath, haemoptysis, fever (body temperature >37.3°C), new onset of moist rales, or new infectious lesion on chest CT; (2) presence of allergic diseases (including but not limited to asthma, allergic rhinitis, allergic purpura, and urticaria), diffuse pan-bronchiolitis, allergic bronchopulmonary aspergillosis (ABPA), Kartagener syndrome, pneumoconiosis, or a history of lung resection and chest wall surgery; (3) presence of malignant tumors, severe cardiac dysfunction, chronic renal failure, or neuromuscular system diseases.

Blood samples and sputum samples were taken from patients with bronchiectasis in each visit. Chronic colonization of pathogen was defined as the presence of two or more times positive culture of the same pathogen from separate sputum samples over at least 2 months.

The study was approved by our hospital's Ethics Committee. All patients provided written informed consent to participate in the present study.

### Methods

Plethysmography, spirometry, and IOS were performed according to the European Respiratory Society (ERS) and the American Thoracic Society (ATS) lung function test guidelines (10). All subjects were offered an opportunity to familiarize themselves with the testing equipment by watching a video and demonstration by technicians and also undergoing a pilot test within 5 days prior to the actual test. The pilot test results were not documented. Patients who used inhalation medication were instructed to discontinue the medication for at least 24 h before the test. On the day of the actual pulmonary function test, reversibility was tested using 400 μg of salbutamol, and the parameters were measured before and after salbutamol administration. The non-force-dependent parameters, the slow lung capacity (SVC) and the force-dependent parameters obtained by IOS were measured in order.

### Plethysmography and Spirometry

The MasterScreen (CareFusion Co, California, US) instrument was used for plethysmography and spirometry. Plethysmography was performed to determine the following lung function parameters: residual volume (RV), total lung capacity (TLC) and RV/TLC ratio. Spirometry was used to determine the following parameters: the vital capacity (VC), forced vital capacity (FVC), forced vital capacity in one second (FEV1), FEV1/FVC ratio, peak expiratory flow (PEF), maximal expiratory flow (MEF) at 75% of the VC (MEF75), MEF at 50% of the VC (MEF50), MEF at 25% of the VC (MEF25), and maximal mid-expiratory flow (MMEF75/25). The baseline lung function level was expressed as the percentage of the predicted value (% pred). Pulmonary function tests were performed in strict accordance with the latest ATS/ERS guidelines (10).

### Impulse Oscillometry

The SentrySuite^®^ IOS system (CareFusion Co, California, US) with SentrySuite^®^ software was used for IOS measurement. The system was calibrated each day before the measurement. The following parameters were collected: central resistance (Rc), peripheral resistance (Rp), total respiratory impedance (Z5), resistance at 5 Hz (R5), resistance at 10 Hz (R10), resistance at 15 Hz (R15), resistance at 20 Hz (R20), resistance at 25 Hz (R25), resistance at 35 Hz (R35), reactance at 5 Hz (X5), and resonant frequency (Fres). The difference between R5–R20, which was an indicator of small airway obstruction, was also calculated.

### Definition of Bronchodilatation Positive

The patients were divided into two subgroups based on their BDT results; a positive and a negative BDT cohort, and subgroup analyses were performed. According to the ATS/ ERS (11) definition, a significant bronchodilator response, i.e., positive BDT was defined as an improvement of ≥12% and ≥200 ml in either FEV1 or FVC after inhalation of bronchodilator compared to the baseline record.

### Bronchiectasis Severity Stratification

A number of scaling systems were adopted to stratify the severity of bronchiectasis, including the Bhalla (12), BRICS (13), modified Reiff (3), Bronchiectasis Severity Index (BSI) (5), and FACED (4) scores. Bhalla score involves nine CT features, and is calculated by subtracting the CT scores from 25, yielding a number that ranges from 3 to 25 (mild 16 to 25; moderate 9 to 15, severe 3 to 8 points). BRICS is a simplified radiological score based on two components: bronchial enlargement and number of bronchopulmonary segments with emphysema. The BRICS score ranges from 0 to 5 points (mild 1 pt; moderate 2 to 3 pts, severe >3 pts). The modified Reiff scores involves the evaluation of the severity of the enlargement of airways by comparing the diameter of the airway with the diameter of the adjacent pulmonary artery, and points are awarded for each lobe (1 = < 2 times, 2 = 2–3 times, 3 = >3 times the diameter of the adjacent pulmonary artery). Since the lingula is treated as a separate lobe, the maximum score is 18 points (mild 0 to 6; moderate 7 to 12, severe 13 to 18 points).

The Bhalla, BRICS and modified Reiff scores are mainly based on the radiological changes of bronchiectasis. The BSI and FACED scores on the other hand incorporate clinical variables with radiological changes to predict clinical outcomes of bronchiectasis. The BSI score incorporates HRCT scores, age, FEV1, dyspnoea, body mass index, exacerbations, hospital admissions and colonization with either *Pseudomonas aeruginosa* or other pathogenic organisms, to produce a score that ranges from 0 to 26 (mild 0 to 4; moderate 5 to 8; severe ≥9 points). Finally, the FACED score incorporates five variables: the FEV1, age, colonization of Pseudomonas, lobe engagement and dyspnoea scores, to produce a number that ranges from 0 to 7 (mild 0 to 2; moderate 3 to 4, severe 5 to 8 points).

We also listed individual parameter such as lobe engagement, type of enlargement, the predominant feature of bronchial wall thickening and enlargement, mucus plugs, atelectasis or lung consolidation, the number of segments with emphysema, as well as the number of bullae.

### Statistical Analysis

Categorical variables were presented as frequencies or percentages, and compared using the Chi-square test (or Fisher's exact test when appropriate). Depending on the distribution, continuous variables were presented as means and standard deviations (SD) when data was normally distributed or as medians with interquartile ranges (IQR) then the distribution was not normal. Comparison between two groups was performed by the Student's *t*-test if the data was normally distributed or Mann–Whitney U-test when the distribution as not normal. The normality of the distribution was tested using the Shapiro-Wilk test. The Kruskal–Wallis test or one-way ANOVA test were used to compare IOS parameters and lung function among patients presenting with different severities of bronchiectasis stratified by the FACED, BSI, modified Reiff, Bhalla and BRICS scores. Spearman's rank correlation coefficient was used for bivariate correlation analysis between IOS parameters, spirometric parameters and bronchiectasis severity scores. IOS parameters that significantly differed between the BDT positive and negative cohorts were then used to predict airway reversibility. Receiver operating characteristic (ROC) curves were performed, and the area under the curve (AUC) of the different IOS parameters was compared using DeLong test (14). For all analyses, *p* < 0.05 were considered statistically significant. All statistical analyses were performed using Stata version 15.1(StataCorp; Texas; USA).

## Results

### Patient Demographics and Basic Clinical Characteristics

A total of 134 patients with bronchiectasis were screened for inclusion, and 91 patients met the inclusion criteria. Seventeen patients were further excluded due to the following concerns: 10 patients did not cooperate during plethysmography, four patients did not undergo the IOS test, and three patients had inaccurate or poor reproducibility of lung function results. Finally, 74 patients completed both the IOS and plethysmography tests (Flow chart in Supplementary Figure 1).

The age of the included subjects ranged from 26 to 81 years, with a mean age of 60.4 ± 11.2 years and 41(55.4%) were males. The mean duration of the disease since diagnosis was 6.7 ± 9.1years. The control group comprised of 121 subjects ranged from 20 to 88 years, with a mean age of 57.5 ± 13.5 years and 68 (56.2%) were males. No statistically significant differences were observed between the bronchiectasis cohort and healthy controls regarding age, gender, and smoking habits (Table 1). Sixteen (21.6%) bronchiectasis had a history of tuberculosis, which is significantly more common than the control group (*p* = 0.01). Thirty-five (47.3%) patients with bronchiectasis had at least one acute exacerbation and 8 (10.8%) patients had more than three exacerbations during the previous year. The sputum culture demonstrated the chronic colonization of *P. aeruginosa* in 11 (14.9%) patients (Table 1).

**Table 1:** Baseline characteristics of bronchiectasis patients and healthy controls.

**Parameters**	**Bronchiectasis patients** ***n* = 74**	**Healthy controls** ***n* = 121**	***P*-value**
**Age, yrs**	60.4 (11.2)	57.5 (13.5)	0.118
**Gender, male**	41 (55.4%)	68 (56.2%)	0.914
**Smoking**			0.293
Non-smoker	44 (59.5%)	81 (66.9%)	
Active smoker	30 (40.5%)	40 (33.1%)	
**Disease years**	6.7 (9.1)	NA	NA
**History of tuberculosis**	16 (21.6%)	7 (5.8%)	0.01
**Comorbidity**			
Hypertension	20 (27.0%)	45 (37.2%)	0.146
Diabetes mellitus	5 (6.8%)	13 (10.7%)	0.353
Chronic heart disease	9 (12.2%)	19 (15.7%)	0.496
**No. of exacerbation**			
≥1	35 (47.3%)	-	-
≥2	19 (25.7%)	-	-
≥3	8 (10.8%)	-	-
**Hospital admission**	10 (13.5%)	NA	NA
**mMRC dyspnea score**		NA	NA
0	2 (2.7%)	-	-
1	33 (44.6%)	-	-
2	25 (33.8%)	-	-
3	11 (14.9%)	-	-
4	3 (4.1%)	-	-
**Sputum culture**		NA	NA
None	57 (77.0%)	-	-
Pseudomonas aeruginosa	11 (14.9%)	-	-
Klebsiella pneumoniae	2 (2.7%)	-	-
Candida albicans	4 (5.4%)	-	-

*Data appeared either with n (%) or mean (SD). mMRC, modified Medical Research Council*.

Most of the included patients had mild to moderate bronchiectasis based on radiological features, with median Bhalla, BRICS and modified Reiff scores of 17, 2 and 5 points, respectively. The BSI and FACED scores which can predict the clinical outcome were 5 and 1 point, respectively, which correspond to mild bronchiectasis. Cylindrical bronchiectasis accounted for 56 (75.7%) of all cases. Some complications of CT features were also evaluated: 26 (35.1%) patients presented with atelectasis or lung consolidation, 21 (28.4%) with mucus plugging, 34 (46.0%) patients presented with emphysema in at least one segment and the presence of bullae were observed in 7 (9.5%) cases (Table 2).

**Table 2:** Severity of bronchiectasis patients based on clinical data and radiological features (*n* = 74).

**Comprehensive** **parameters**	**Bronchiectasis** **patients**	**Individual** **parameter**	**Bronchiectasis** **patients**
**Bhalla score, median (IQR)**	17.0 (14.0, 19.0)	**No. of affected lobes**	4 (2, 5)
**Bhalla stratification**		**Type**	
Mild	49 (66.2%)	cylindrical	56 (75.7%)
Moderate	23 (31.1%)	cystic	18 (24.3%)
Severe	2 (2.7%)	**Bronchial wall thickening**	
**BRICS score, median (IQR)**	2.0 (1.0, 3.0)	slight	17 (23.0%)
**BRICS stratification**		moderate	44 (59.5%)
Mild	25 (33.8%)	severe	13 (17.6%)
Moderate	39 (52.7%)	**Bronchial dilatation grade**	
Severe	10 (13.5%)	slight	42 (56.8%)
**Reiff score, median (IQR)**	5.0 (3.0, 8.0)	moderate	16 (21.6%)
**Reiff stratification**		severe	16 (21.6%)
Mild	47 (63.5%)	**Atelectasis or consolidation**	
Moderate	18 (24.3%)	Yes	26 (35.1%)
Severe	9 (12.2%)	No	48 (64.9%)
**BSI score, median (IQR)**	5.0 (3.0, 8.0)	**With mucus plugs**	21 (28.4%)
**BSI stratification**		**No. of segments with emphysema**	
Mild	36 (48.6%)	0	40 (54.1%)
Moderate	27 (36.5%)	1	27 (36.5%)
Severe	11 (14.9%)	2	7 (9.5%)
**FACED score, median (IQR)**	1.0 (1.0, 3.0)	**No. of bullae**	
**FACED stratification**		0	67 (90.5%)
Mild	48 (64.9%)	1	3 (4.1%)
Moderate	18 (24.3%)	2	1 (1.4%)
Severe	8 (10.8%)	3	3 (4.1%)

*Categorical data appeared as n (%), continuous variable expressed with median (IQR). Left part, comprehensive parameter, right part, individual parameters*.

### IOS Parameters Differ in Different Bronchiectasis Severity Groups Stratified by Multidimensional Scores Based on Both Radiology and Clinical Characteristics

**Figure 1:**
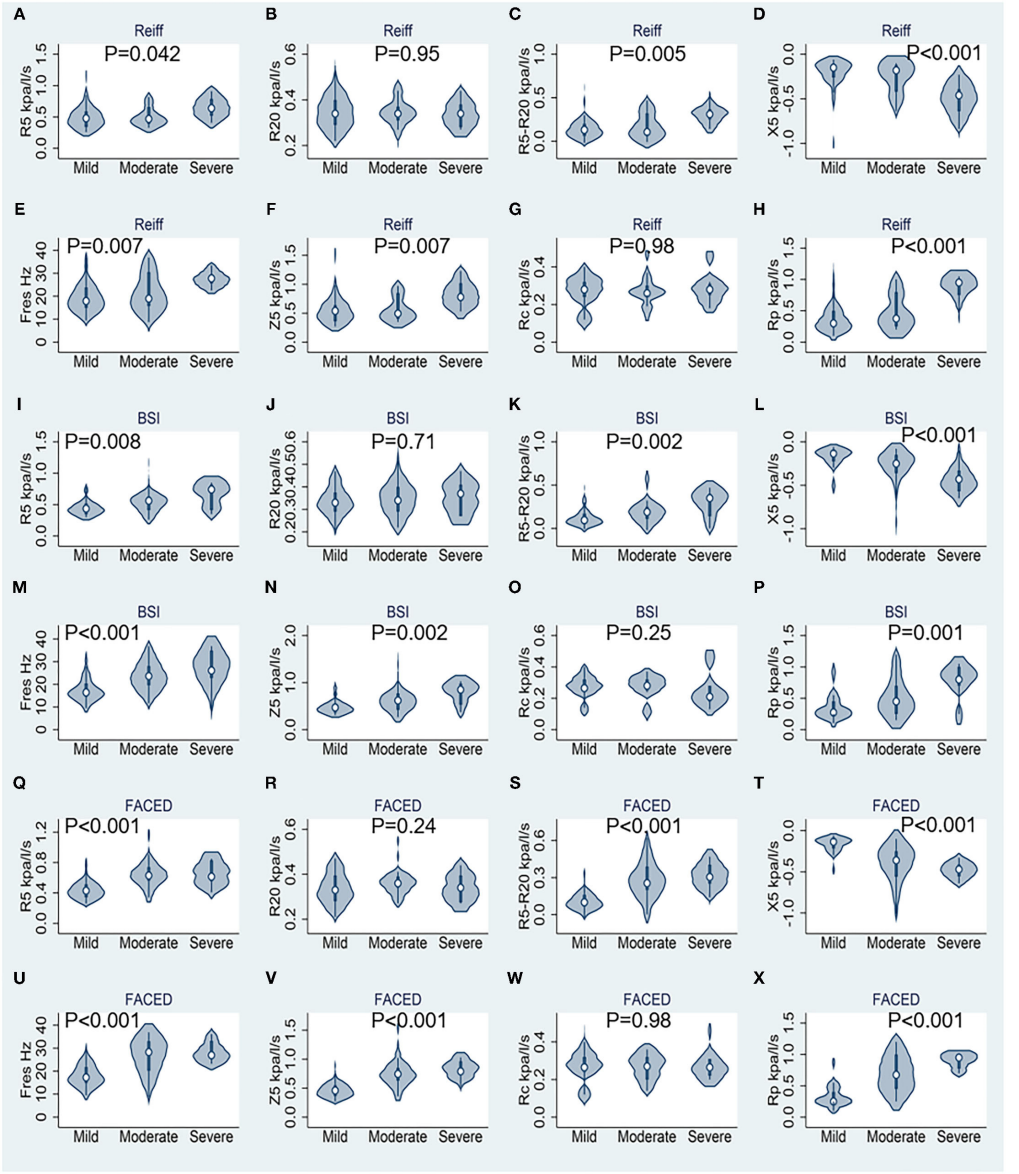
Results of respiratory impedance at rest stratified by different bronchiectasis severity scores (*n* = 74). **(A–H)** Correspond to the modified Reiff score, **(I–P)** correspond to the BSI score, and **(Q–X)** correspond to the FACED score. R5, respiratory system resistance at 5 Hz; R20, respiratory system resistance at 20 Hz; X5, respiratory system reactance at 5 Hz; Fres, resonant frequency; Z5, respiratory impedance; Rc, central resistance; and Rp, peripheral resistance. Statistics performed by Kruskal–Wallis test.

Correlation between IOS parameters and the modified Reiff, BSI and FACED scores are shown in Figure 1. The results demonstrated that as the severity of bronchiectasis increased, IOS parameters R5, R5-R20, Fres, Z5 and Rp had the tendency to increase, while the X5 had the tendency to decreased in value. All these IOS parameters significantly differed among the three different bronchiectasis severity cohorts except for R20 and the Rc. Similar results were observed regarding the BSI and FACED scores (Figure 1).

With regard to the lung function measured by spirometry and plethysmography, patients with a mild grade of bronchiectasis had best FVC, FEV1 and FEV1/FVC ratio, compared with those who had moderate and severe bronchiectasis (*p* < 0.001 for all variables) according to FACED, modified Reiff and BSI scores. Air-trapping markers RV and RV/TLC ratio significantly increased in patients with moderate and severe bronchiectasis than the mild ones, regardless of the severity scale used (FACED, modified Reiff or BSI scores). While the MEF75, MEF50, MEF25, MMEF75/25, as well as PEF all decreased as the bronchiectasis severity increased according to the FACED score (Supplementary Tables 1–5).

Mutual comparison between the mild, moderate, and severe cohorts of bronchiectasis in terms of different evaluation scales and detailed IOS parameters, spirometry and plethysmography data were shown in Supplementary Tables 1–5. Compared with patients with mild disease, those who presented moderate to severe disease had poorer lung function and significant increase in airway resistance. However, because of the small sample size of this study, difference between moderate and severe group did not reach statistical significance in some parameters.

### IOS Parameters Predict Airway Reversibility

As shown in Table 3, bronchiectasis patients had higher airway resistance (Rc, Rp, Z5, R5, R20, and R5-R20) and airway reactance (X5 and Fres) on IOS, compared to health controls. The differences were statistically significant (*p* < 0.001). Spirometry parameters FVC, FEV1, and FEV1/FVC ratio were also lower in bronchiectasis patients compared to health controls (*p* < 0.001). RV and RV/TLC ratio were significantly increased in the bronchiectasis group (*p* < 0.001). MEF75, MEF50, MEF25, and MMEF75/25 were significantly decreased in bronchiectasis (*p* < 0.001 for all variables).

**Table 3:** Impulse oscillometry and spirometry data of patients with bronchiectasis and controls, and between the bronchodilator positive and negative cohort.

**Parameters**	**Bronchiectasis vs. control**			**Bronchiectasis (BE) patients (*****n*** **=** **74)**		
	**Non-BE control** **(*n* = 121)**	**BE patients** **(*n* = 74)**	***p*-value**	**BDT negative** **(*n* = 53)**	**BDT positive** **(*n* = 21)**	***p*-value**
**Rc, kpa/l/s**	0.2 (0.1–0.3)	0.3 (0.2–0.3)	<0.001	0.3 (0.2–0.3)	0.3 (0.2–0.3)	0.91
**Rp, kpa/l/s**	0.3 (0.1–0.3)	0.4 (0.3–0.7)	<0.001	0.3 (0.3–0.5)	0.7 (0.4–0.9)	<0.001
**Z5, kpa/l/s**	0.3 (0.3–0.4)	0.5 (0.4–0.7)	<0.001	0.5 (0.4–0.6)	0.7 (0.6–0.9)	<0.001
**R5, kpa/l/s**	0.3 (0.3–0.4)	0.5 (0.4–0.6)	<0.001	0.5 (0.4–0.5)	0.6 (0.5–0.8)	<0.001
**R20, kpa/l/s**	0.2 (0.3–0.4)	0.3 (0.3–0.4)	<0.001	0.3 (0.3–0.4)	0.3 (0.3–0.4)	0.34
**R5-R20, kpa/l/s**	0.0 (0.0–0.1)	0.1 (0.1–0.3)	<0.001	0.1 (0.1–0.2)	0.3 (0.2–0.4)	<0.001
**X5, kpa/l/s**	−0.1 (−0.1–−0.1)	−0.2 (−0.4–−0.1)	<0.001	−0.1 (−0.3–−0.1)	−0.4 (−0.5–−0.3)	<0.001
**Fres, Hz**	11.2 (9.5–14.4)	20.0 (15.4–25.6)	<0.001	17.5 (15.2–23.4)	25.7 (19.9–30.6)	<0.001
**FEV1, % pred**	96.7 (13.4)	70.4 (27.1)	<0.001	78.5 (25.7)	49.9 (18.5)	<0.001
**FVC, % pred**	100.1 (15.6)	82.9 (20.9)	<0.001	86.4 (21.6)	74.1 (16.0)	0.021
**FEV1/FVC**	81.2 (8.4)	66.8 (15.8)	<0.001	72.3 (13.7)	53.3 (12.2)	<0.001
**RV, % pred**	100.7 (83.9–115.3)	126.7 (101.0–165.1)	<0.001	117.4 (96.5–137.8)	171.9 (138.3–196.1)	<0.001
**TLC, % pred**	97.3 (88.5–108.5)	99.2 (88.9–112.3)	0.27	97.6 (85.5–106.2)	106.7 (101.5–118.4)	0.002
**RV/TLC**	33.4 (29.2–39.1)	47.3 (42.0–60.6)	<0.001	45.2 (40.5–53.9)	60.3 (49.1–72.7)	<0.001
**MEF75, % pred**	98.4 (83.4–108.7)	55.6 (21.8–88.1)	<0.001	71.1 (50.3–96.6)	18.5 (12.8–37.3)	<0.001
**MEF50, % pred**	81.7 (64.3–94.9)	40.6 (18.9–61.0)	<0.001	48.2 (34.4–69.0)	17.2 (9.3–26.8)	<0.001
**MEF25, % pred**	63.9 (45.6–81.2)	27.7 (17.7–53.5)	<0.001	38.5 (23.3–57.6)	18.0 (13.9–23.4)	<0.001
**MMEF, % pred**	74.9 (57.9–91.1)	35.6 (18.4–56.3)	<0.001	44.7 (31.3–65.3)	17.6 (11.0–24.6)	<0.001
**PEF, % pred**	101.5 (93.8–111.4)	73.9 (45.1–99.9)	<0.001	87.3 (71.6–101.3)	47.3 (33.1–59.0)	<0.001
**VC IN, % pred**	88.9 (76.7–101.3)	66.9 (51.2–81.8)	<0.001	67.5 (57.2–87.0)	60.6 (48.3–78.3)	0.16

*Data presented as mean (SD) or median (IQR) as appropriate; Rc, central resistance; Rp, peripheral resistance; Z5, respiratory impedance at 5 Hz; R5 and R20, respiratory system resistance at 5 and 20 Hz, respectively; X5, respiratory system reactance at 5 Hz; Fres, resonant frequency; FEV1, forced expiratory volume in one second; FVC, forced vital capacity; RV, residual volume; TLC, total lung capacity; MEF, maximal expiratory flow; MMEF, maximal mid-expiratory flow; PEF, peak expiratory flow; VC IN, inspiratory vital capacity*.

**Figure 2:**
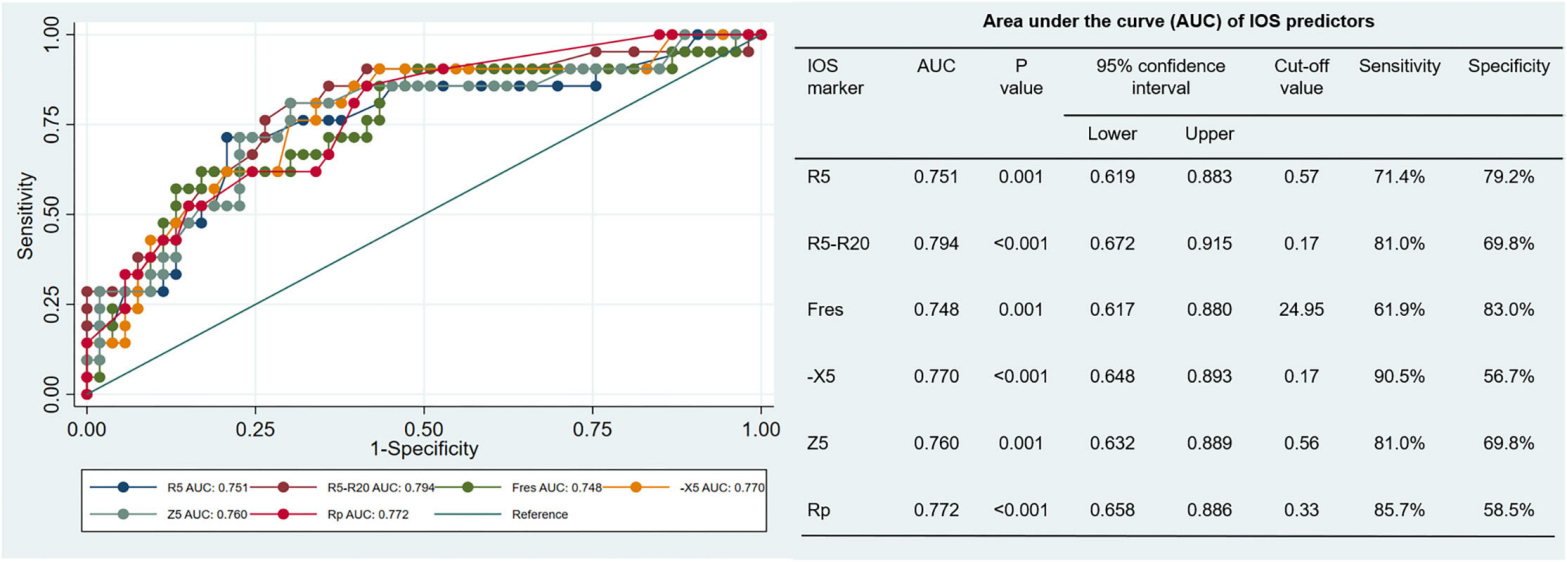
Receiver Operator Characteristic (ROC) curve: x-axis (1-specificity), y-axis sensitivity, each curve represents the predictability of each impulse oscillometry (IOS) parameter (in different colors). AUC values and *p*-values for the separate variables are detailed in the right table. R5, respiratory system resistance at 5 Hz; R20, respiratory system resistance at 20 Hz; Fres, resonant frequency; X5, respiratory system reactance at 5 Hz; Z5, respiratory impedance, and Rp, peripheral resistance.

Compared with the BDT negative subgroup, all IOS parameters other than the Rc and Rp increased in the BDT positive subgroup. The diagnostic values of the previously selected IOS parameters in airway reversibility were shown in Figure 2. The AUC and 95% confidence interval for R5, R5-R20, Fres, -X5, Z5 and Rp were 0.751 (0.619–0.883), 0.794 (0.672–0.915), 0.748 (0.617–0.880), 0.77 (0.648–0.893), 0.76 (0.632–0.889) and 0.772 (0.658–0.886), respectively. Among these markers, R5-R20 best predicted the BDT response, with a sensitivity of 81.0% and specificity of 69.8%, followed by Rp, -X5, Z5, R5 and the Fres (Figure 2). Delong test revealed no statistically significant difference among the AUC of the six parameters explored. The results demonstrated that value of R5-R20 was useful for predicting airway reversibility in patients with bronchiectasis. Since R5 in general represents the total airway resistance, and R20 represents the central and large airway resistance, the difference of R5-R20 infers the peripheral airway resistance. Although the predictive usefulness was almost equivalent among above IOS biomarkers, the cut-off value of R5-R20 for predicting airway reversibility was superior to other indicators.

### IOS Parameters Correlate Well With Bronchiectasis Severity Scores and Lung Function Parameters

**Figure 3:**
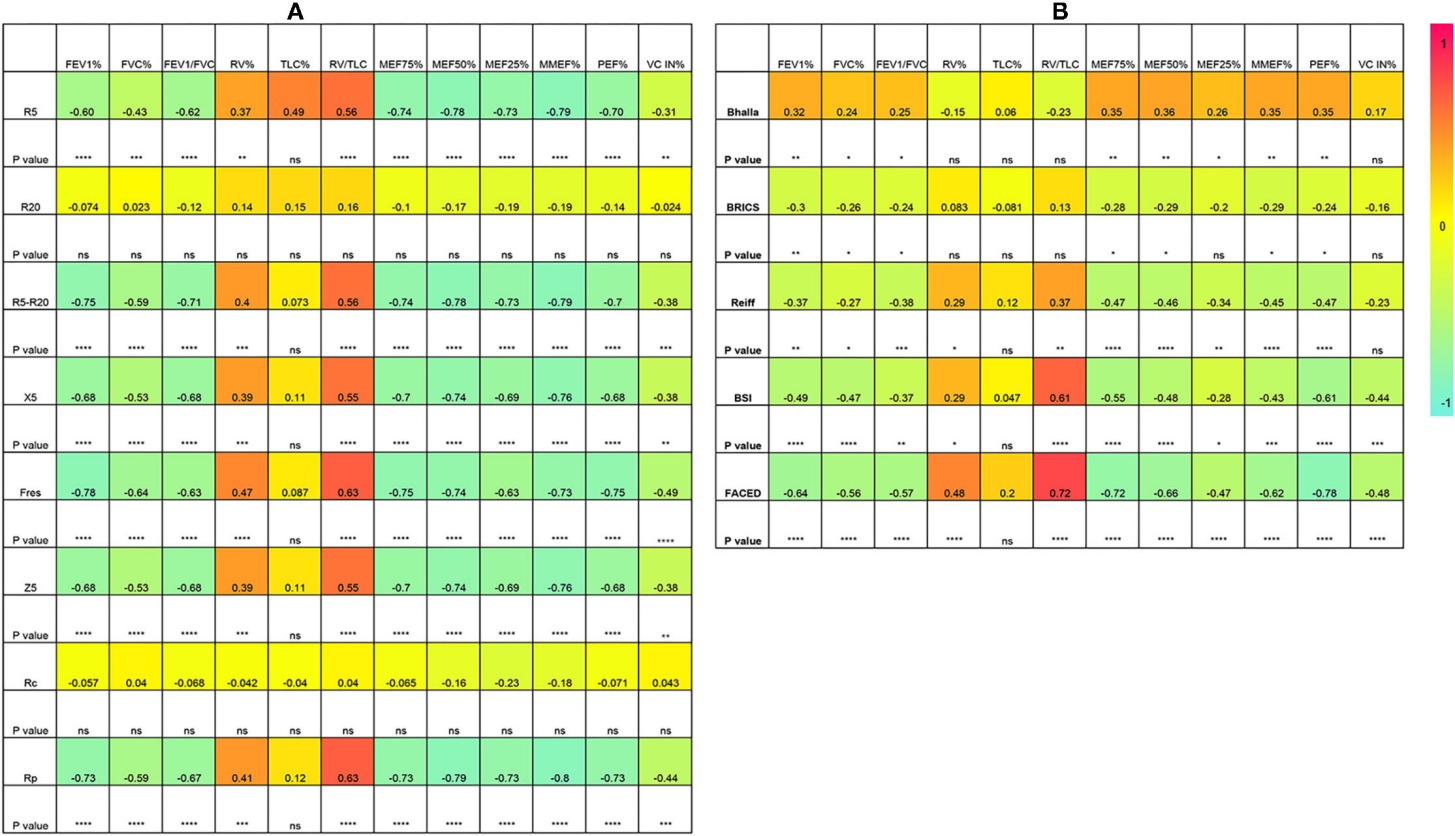
Spearman correlation analysis. **(A)** between IOS parameters and lung function; **(B)** Between lung function and bronchiectasis severity scores. ^*^p < 0.05; ^**^p < 0.01; ^***^p < 0.001; ^****^p < 0.0001; ns, no significance. Rc, central resistance; Rp, peripheral resistance; Z5, the respiratory impedance; R5 and R20, respiratory system resistance at 5 and 20Hz, respectively; X5, respiratory system reactance at 5Hz; Fres, resonant frequency. FEV1, forced expiratory volume in one second; FVC, forced vital capacity; RV, residual volume; TLC, total lung capacity; MEF, maximal expiratory flow; MMEF, maximal mid-expiratory flow; PEF, peak expiratory flow.

Of all the IOS parameters, R5, R5-R20, X5, Fres, Z5 and Rp showed a negative correlation with the spirometry parameters FEV1, FVC, FEV1/FVC ratio. RV and RV/TLC ratio correlated positively with all the IOS parameters mentioned above. MEF75, MEF50, MEF25, and MMEF75/25 correlated negatively with respiratory reactance and resistance, but not R20 and Rc. A strong correlation was observed between the two indicators of small airway dysfunction, the frequency dependence of resistance (FDR, R5-20) and MMEF75/25 (rs = −0.79, *p* < 0.0001). Fres demonstrated the strongest correlation with FEV1 (rs = −0.78, *p* < 0.0001), followed by FDR (rs = −0.75, *p* < 0.0001) (Figure 3).

Regarding the correlation between bronchiectasis severity scores and lung function parameters, results showed that FEV1, FVC, FEV1/FVC ratio correlated negatively with the BRICS, modified Reiff, BSI and FACED scores. As low Bhalla scores reflected high severity, a positive correlation was demonstrated between the Bhalla score and lung function parameters. Similarly, MEF75, MEF50, MEF25, MMEF75/25 were also negatively associated with bronchiectasis severity scores. The RV/TLC ratio showed a moderate correlation with BSI scores (rs = 0.61, *p* < 0.0001) and a strong correlation with FACED scores (rs = 0.72, *p* < 0.0001) (Figure 3).

**Figure 4:**
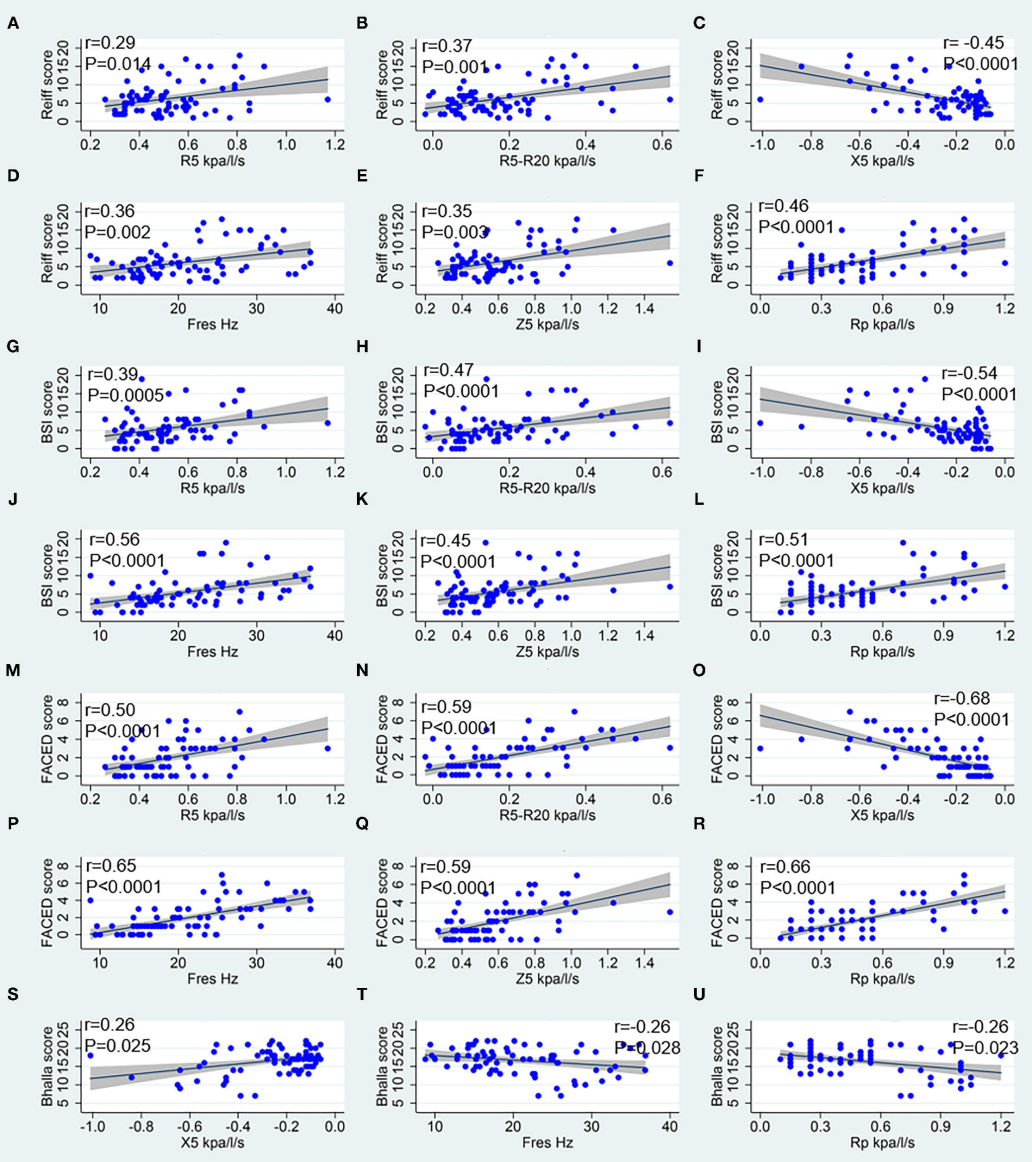
Spearman correlation analysis between IOS parameters and bronchiectasis severity scores. **(A–F)** Correspond to the Reiff score, **(G–L)** correspond to the BSI score, **(M–R)** correspond to the FACED score, and **(S–U)** correspond to the Bhalla score. R5, respiratory system resistance at 5 Hz; R20, respiratory system resistance at 20 Hz; X5, respiratory system reactance at 5 Hz; Fres, resonant frequency; Z5, the respiratory impedance; Rc, central resistance; and Rp, peripheral resistance.

**Figure 5:**
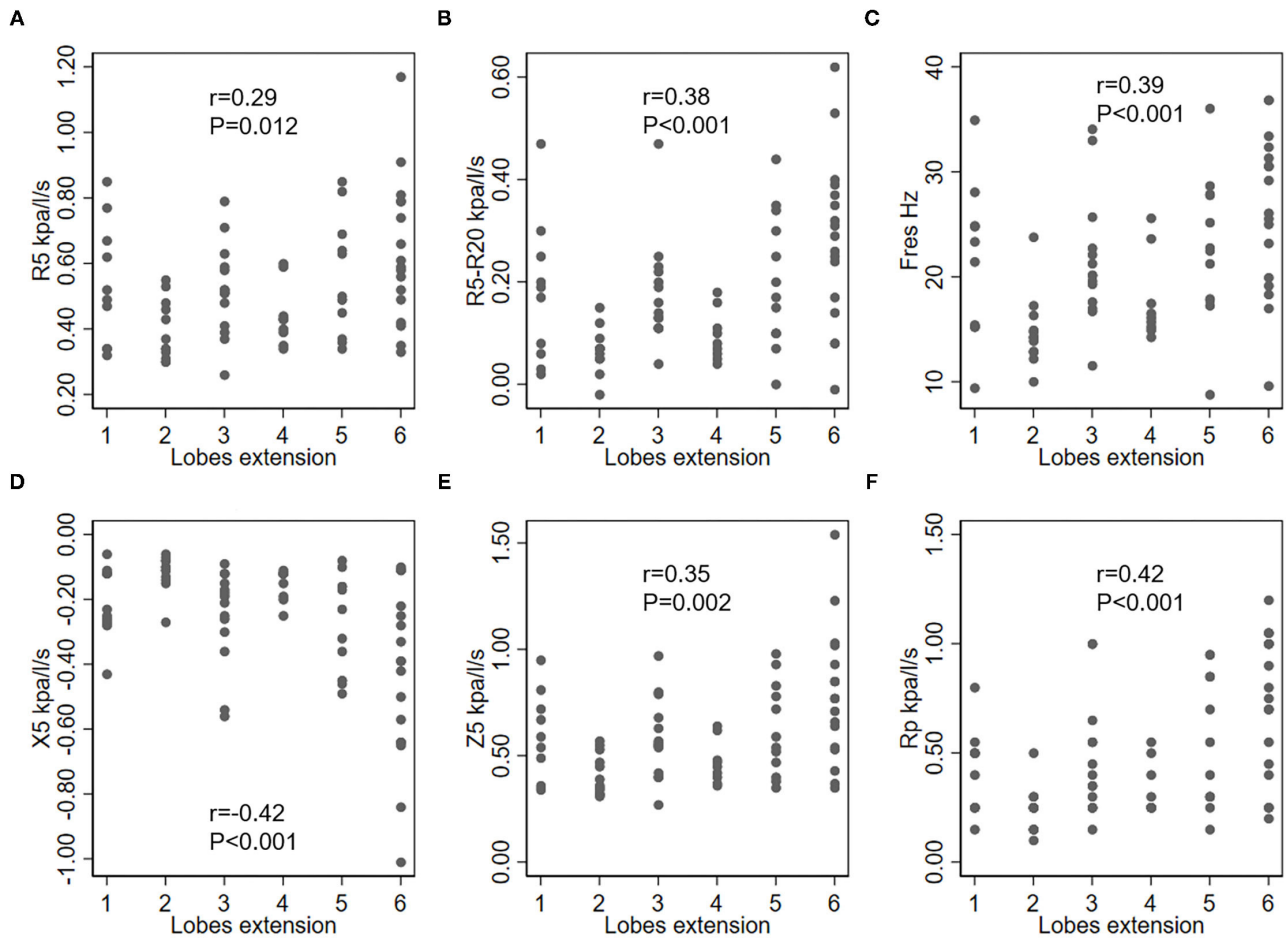
Correlation between IOS parameters and lobe engagement (with the lingula counted as an individual lobe). **(A)** R5, respiratory system resistance at 5 Hz; **(B)** R5–R20, difference between the respiratory system resistance at 5 Hz and 20 Hz; **(C)** Fres, resonant frequency; **(D)** X5, respiratory system reactance at 5 Hz; **(E)** Z5, the respiratory impedance; **(F)** Rp, peripheral resistance.

While considering the correlation between IOS parameters and bronchiectasis severity scores, the modified Reiff scores correlated positively with R5, R5-R20, Fres, Z5, and Rp, and negatively with X5. A similar correlation was observed between BSI scores and IOS parameters. Compared with both the modified Reiff and BSI scores, the FACED scores showed moderate correlation with IOS parameters. However, only a few IOS parameters (X5, Fres and Rp) correlated with Bhalla scores. No correlation between IOS parameters and BRICS scores was detected (data not shown). Overall, we noticed that the airway reactance (X5 and Fres) showed a stronger correlation with BSI scores than airway resistance. The modified Reiff and FACED scores, on the other hand, showed stronger correlation with Rp than airway reactance measurements (X5 and Fres) (Figure 4). In addition, the number of bronchiectasis lobes showed a weak correlation with IOS parameters R5, R5-R20, Fres, X5, Z5, and Rp (Figure 5).

### IOS Parameters Do Not Correlate With Pseudomonas Infection or Hospital Admission

As shown in Table 4, patients chronically colonized with Pseudomonas had longer disease duration (median: 12.5 vs. 3.0 years, *p* < 0.001). Acute exacerbation (*p* = 0.005) and hospital admission (*p* = 0.017) occurred more frequently in the Pseudomonas positive subgroup than the negative. Bronchiectasis patients with chronic Pseudomonas infection also presented lower FVC, VC, and PEF than the Pseudomonas negative subgroup. However, there were no significant differences in IOS parameters between Pseudomonas positive and negative subgroups Patients with previous hospital admission had longer disease duration and had a higher number of acute exacerbations than the hospital admission free group. Nevertheless, there were no significant differences in IOS parameters between patients with previous hospital admission and those without, although the traditional lung parameters FVC, FEV1 significantly decreased in patients with previous hospital admission (Table 5).

**Table 4:** Comparison between bronchiectasis patients in terms of the chronic colonization of Pseudomonas aeruginosa.

**Parameters**	**Pseudomonas negative** **(*n* = 63)**	**Pseudomonas positive** **(*n* = 11)**	***p*-value**
**Age, yrs**	60.5 (10.5)	59.7 (15.5)	0.83
**Gender**			0.47
Male	36 (57.1%)	5 (45.5%)	
Female	27 (42.9%)	6 (54.5%)	
**BMI, kg·m** ^**−2**^	23.0 (4.6)	20.5 (4.1)	0.10
**Disease years**	3.0 (1.0, 5.0)	12.5 (5.0, 18.0)	<0.001
**Exacerbation**	0.0 (0.0, 1.0)	2.0 (1.0, 3.0)	0.005
**Hospital admission**	0.0 (0.0, 0.0)	0.0 (0.0, 1.0)	0.017
**mMRC score**			0.051
**0**	2 (3.2%)	0 (0.0%)	
**1**	31 (49.2%)	2 (18.2%)	
**2**	21 (33.3%)	4 (36.4%)	
**3**	8 (12.7%)	3 (27.3%)	
**4**	1 (1.6%)	2 (18.2%)	
**IOS and lung function parameters**			
**Rc, kpa/l/s**	0.3 (0.2, 0.3)	0.3 (0.3, 0.3)	0.15
**Rp, kpa/l/s**	0.3 (0.3, 0.6)	0.7 (0.3, 0.9)	0.060
**Z5, kpa/l/s**	0.5 (0.4, 0.7)	0.6 (0.5, 0.8)	0.11
**R5, kpa/l/s**	0.5 (0.4, 0.6)	0.6 (0.4, 0.6)	0.22
**R20, kpa/l/s**	0.3 (0.3, 0.4)	0.4 (0.3, 0.4)	0.64
**R5-R20, kpa/l/s**	0.1 (0.1, 0.3)	0.2 (0.1, 0.3)	0.22
**X5, kpa/l/s**	−0.2 (−0.3, −0.1)	−0.3 (−0.5, −0.2)	0.056
**Fres, Hz**	18.3 (15.3, 25.2)	23.2 (20.2, 26.1)	0.14
**FEV1, % pred**	72.5 (27.0)	57.9 (25.5)	0.098
**FVC, % pred**	85.0 (19.8)	71.3 (23.8)	0.044
**FEV1/FVC**	67.0 (15.7)	65.9 (17.0)	0.83
**RV, % pred**	130.0 (98.5, 165.3)	121.2 (107.9, 157.9)	0.68
**TLC, % pred**	100.1 (87.4, 112.4)	93.4 (88.9, 105.0)	0.27
**RV/TLC**	46.8 (41.3, 60.5)	53.9 (46.7, 64.1)	0.085
**MEF75, % pred**	58.7 (22.6, 94.9)	37.7 (17.7, 57.8)	0.11
**MEF50, % pred**	44.4 (18.9, 64.8)	31.1 (12.0, 46.0)	0.21
**MEF25, % pred**	28.0 (18.4, 53.2)	25.5 (14.2, 55.1)	0.67
**MMEF, % pred**	36.5 (19.4, 61.4)	29.1 (12.2, 42.5)	0.30
**PEF, % pred**	77.9 (47.3, 100.4)	56.2 (32.0, 74.4)	0.034
**VC IN, % pred**	68.5 (57.2–87.0)	58.8 (29.7–75.1)	0.039

*Rc, central resistance; Rp, peripheral resistance; Z5, respiratory impedance at 5 Hz; R5 and R20, respiratory system resistance at 5 and 20 Hz, respectively; X5, respiratory system reactance at 5 Hz; Fres, resonant frequency; FEV1, forced expiratory volume in one second; FVC, forced vital capacity; RV, residual volume; TLC, total lung capacity; MEF, maximal expiratory flow; MMEF, maximal mid-expiratory flow; PEF, peak expiratory flow; VC IN, inspiratory vital capacity*.

**Table 5:** Comparison between bronchiectasis patients with and without hospital admission.

**Parameters**	**No hospital admission** **(*n* = 64)**	**Hospital admission** **(*n* = 10)**	***p*-value**
**Age, yrs**	59.6 (11.5)	65.3 (8.7)	0.14
**Gender**			0.32
Male	34 (53%)	7 (70%)	
Female	30 (47%)	3 (30%)	
**BMI, kg·m** ^**−2**^	23.1 (4.5)	19.5 (3.9)	0.017
**Disease years**	3.0 (1.0–5.5)	13.5 (3.0–20.0)	0.020
**Exacerbation**	0.0 (0.0–1.0)	2.0 (1.0–3.0)	0.002
**mMRC**			<0.001
0	2 (3%)	0 (0%)	
1	33 (52%)	0 (0%)	
2	21 (33%)	4 (40%)	
3	7 (11%)	4 (40%)	
4	1 (2%)	2 (20%)	
**IOS and lung function parameters**			
**Rc, kpa/l/s**	0.3 (0.2–0.3)	0.2 (0.2–0.3)	0.18
**Rp, kpa/l/s**	0.3 (0.3–0.6)	0.8 (0.3–1.0)	0.094
**Z5, kpa/l/s**	0.5 (0.4–0.7)	0.7 (0.4–0.9)	0.21
**R5, kpa/l/s**	0.5 (0.4–0.6)	0.6 (0.4–0.8)	0.43
**R20, kpa/l/s**	0.3 (0.3–0.4)	0.3 (0.3–0.4)	0.21
**R5-R20, kpa/l/s**	0.1 (0.1–0.2)	0.3 (0.1–0.4)	0.074
**X5, kpa/l/s**	−0.2 (−0.3–0.1)	−0.4 (−0.6–0.1)	0.067
**Fres, Hz**	19.5 (15.3–25.1)	24.4 (18.3–29.2)	0.13
**FEV1, % pred**	73.1 (25.8)	52.8 (30.1)	0.026
**FVC, % pred**	86.0 (18.8)	63.4 (23.6)	0.001
**FEV1/FVC**	67.2 (14.8)	64.2 (21.6)	0.58
**RV, % pred**	128.2 (100.8–164.3)	124.1 (101.0–180.0)	0.89
**TLC, % pred**	99.7 (90.6–112.5)	92.5 (83.2–104.5)	0.12
**RV/TLC**	46.8 (41.7–59.0)	63.5 (46.8–72.1)	0.060
**MEF75, % pred**	57.8 (27.2–89.6)	25.6 (10.1–88.0)	0.082
**MEF50, % pred**	43.0 (20.3–62.3)	20.1 (8.3–46.7)	0.12
**MEF25, % pred**	27.6 (18.5–53.3)	43.8 (13.0–53.5)	0.72
**MMEF, % pred**	36.5 (20.8–58.9)	21.6 (8.3–48.9)	0.18
**PEF, % pred**	77.3 (49.2–100.0)	36.6 (27.4–88.8)	0.060
**VC IN, % pred**	67.5 (56.3–85.3)	59.7 (29.7–75.1)	0.072

*Rc, central resistance; Rp, peripheral resistance; Z5, respiratory impedance at 5 Hz; R5 and R20, respiratory system resistance at 5 and 20 Hz, respectively; X5, respiratory system reactance at 5 Hz; Fres, resonant frequency; FEV1, forced expiratory volume in one second; FVC, forced vital capacity; RV, residual volume; TLC, total lung capacity; MEF, maximal expiratory flow; MMEF, maximal mid-expiratory flow; PEF, peak expiratory flow; VC IN, inspiratory vital capacity*.

## Discussion

Results of the present study supported previous findings that IOS parameters correlated with the severity of bronchiectasis assessed by scoring tools that incorporate clinical characteristics, such as the FACED and BSI scores. Both the airway resistance and reactance tend to increase as bronchiectasis severity advanced. Furthermore, the present study explored the correlation between IOS parameters and airway reversibility. A large proportion of patients (28%) in the cohort showed positive responses to the β2 agonist salbutamol. IOS parameter R5-R20 was found to best predict BDT response. To the best of our knowledge, this was the first study that demonstrated the usefulness of IOS in the assessment of airway reversibility in non-cystic fibrosis bronchiectasis.

Bronchiectasis is a heterogenous disease. Many factors contribute to the development of bronchiectasis, including the early infection by Mycobacteria tuberculosis and chronic colonization by microorganism with potential pathogenicity (MPP) (2). In the present study, 21.6% of the recruited bronchiectasis patients presented with a history of tuberculosis infection. Airway pathogens were isolated in 23% of patients, and *P. aeruginosa* was found to be the major MMP in our study. Infections are a common cause of bronchiectasis. In a Greek study, previous infection (25.2%) and previous tuberculosis (TB) (22.3%) were the most commonly identified underlying conditions in bronchiectasis (15). Data from a recent large cohort study suggests that previous infection was the ethology of bronchiectasis in 40.4% of the cases in the Caucasian population. The presence of TB was observed in 13.5% of the patients, while 25.6% of these patients had chronic infection related to *P. aeruginosa* (16). Another study in a Taiwanese population indicated that 12% of bronchiectasis cases could be attributed to an early infection of Mycobacteria tuberculosis (17). The apparent differences in percentage could be explained by geographical differences and relatively small sample sizes. Interestingly, in the European study, the presence of Hemophilus influenzae and *P. aeruginosa* were identified as the most common pathogens, while in the US, non-tuberculous mycobacteria (NTM) and *P. aeruginosa* were found to be relatively common, while Hemophilus influenzae were less often detected in bronchiectasis (5, 18).

Bronchiectasis is a heterogeneous disease etiologically involving diverse factors. Therefore, no single parameter can be used to determine its overall severity and prognosis. Bronchial enlargement, bronchial wall thickening, and other individual features of bronchiectasis could be easily recognized on a HRCT scan of the thorax. However, systematic quantification of these abnormalities could be more difficult. In this study, we use multidimensional scoring tools to stratify the severity of bronchiectasis into mild, moderate, and severe. As mentioned above, the FACED and BSI scores are two parameters well established and validated to predict future outcomes. FACED is concerned about patients being at low, moderate and high risk of death. While BSI scores contain more variables like BMI, exacerbations, hospital admissions, etc., therefore focus on the quality of life. Airway structures and morphology assessment was mainly interpreted by the modified Reiff score. Accurate stratification of bronchiectasis could allow clinicians to take relevant preventive measures and prescribe adequate treatment and medication (19, 20). In the present study, 85.1% of patients were categorized into mild (48.6%) and moderate (36.5%) bronchiectasis according to the BSI (5) scores, which mean that the mortality and hospitalization rate in 4 years were predicted to rise to 11.3 and 19.4%, respectively (19). Similar results are obtained using the FACED (4) scores, where 89.2% were categorized into mild (64.9%) and moderate (24.3%) bronchiectasis. Patients with mild and moderate disease severity according to FACED were predicted to have a 5-year mortality of 4 and 25%, respectively (19). The modified Reiff (3) score, which could better reflect airway damages instead categorized 87.8% of the included patients as mild or moderate disease. These results were in line with the study by Radovanovic et al. (21), and consistent with the results using FACED and BSI scores. Complication such as atelectasis and lung consolidation are not uncommon in bronchiectasis. In the present study, more than one-third of the patients showed atelectasis or lung consolidation on CT scan. In cystic fibrosis patients, such abnormalities might be considered a biomarker that predicts later development of bronchiectasis if detected in childhood (22).

Airway reversibility tests showed that 21 out of 74 (28%) bronchiectasis patients in the present study were BDT positive. The British Thoracic Society guideline (20) for non-cystic fibrosis bronchiectasis recommends reversibility testing to identify co-existing obstructive conditions. An Australian study suggested that 17% of hospitalized bronchiectasis patients had bronchodilator reversibility in their large airways (FEV1). Furthermore, 41% of the subjects also showed reversibility in their small airways. However, the study population was relatively small and many patients presented with asthma or COPD (23). However, after excluding all patients with comorbid asthma and COPD, another study on patients with stable bronchiectasis still demonstrated a large proportion with positive BDT (24). Previous studies had shown that BDT could be positive in up to 25% of patients with bronchiectasis (18, 24, 25). This may be explained: firstly, by the changes in the structures of airways. Secondly, chronic airway infection may lead to airway spasms that temporally affect the airway mechanics.

Spirometry, which is currently utilized in the clinic for measurement of lung function primarily reflect airflow characteristics, and the results depend on the central airway diameter, lung volume, lung elastic retraction force, respiratory muscle strength, and forced expiratory pattern (26). Spirometry parameters like FEV1 and FVC, which are commonly used to assess the degree of airway obstruction in obstructive pulmonary diseases such as asthma and COPD has inherent limitations when used to evaluate the severity of bronchiectasis because the pathophysiological mechanisms of bronchiectasis are different from those of obstructive lung disease. In contrast, IOS mainly evaluates the airway diameter and measures small airway function. Compared to traditional lung function tests, the performance of IOS shows two main advantages: firstly, IOS is a passive method that does not require forced exhalation and is not related to respiratory muscle strength. Secondly, this measurement adopts a series of wave frequencies that can reach different airway subsegments, giving a comprehensive report including the airway resistance, reactance and the resonant frequency for both inspiration and expiration, thus providing important information about regional heterogeneity and lung periphery. The results of the present study show that IOS parameters that reflect airway resistance and reactance present a tendency to increase based on the severity of bronchiectasis, suggesting that IOS can be useful in the assessment of bronchiectasis severity. Our data are consistent with the results by Yamamoto et al. (9). Besides FACED and BSI scores, we also validated IOS's usefulness in assessing the severity of bronchiectasis stratified by predominately radiological scoring system such as the modified Reiff score. Our results confirmed the value of IOS parameters in differentiating the degree of radiological airway changes. However, in our study, the Rc and R20 were not statistically significant among the different bronchiectasis severity subgroups. Notably, these two parameters mainly measure central airway resistance. These results suggest that the degree of central airway obstruction is similar in bronchiectasis regardless of disease severity. In summary, IOS parameters correlate well with both radiographic and clinical severity of bronchiectasis.

In the present study, bronchiectasis patients with positive BDT had decreased FEV1 and FEV1/FVC ratio on spirometry, demonstrating an obstructive ventilatory dysfunction. When we look at the IOS parameters, the small airway resistance indicator R5-R20 increased, but the large airway resistance indicators Rc and R20 remain the same. This suggested that airway obstruction in bronchiectasis mainly occurs in peripheral airways and not central ones. This could be further confirmed by the decrease of the MEF25 and MMEF75/25, parameters reflecting the changes in the small airways' flow rate. In addition, the airway trapping markers RV and RV/TLC ratio increased in bronchiectasis. The pathophysiological features of bronchiectasis are heterogeneous, both obstructive and restrictive ventilation dysfunction may present. However, a multicenter, prospective and observational study suggests that airway trapping and lung diffusing capacity impairment are the most common lung function abnormalities in bronchiectasis (21).

ROC curves were drawn to find out which IOS parameter best predicted airway reversibility in bronchiectasis. The AUC of IOS parameters indicated that the small airway resistance indicator R5-R20 was superior to other IOS parameters in differencing between patients with positive BDT and negative BDT. In bronchiectasis, airflow obstruction was found to be predominantly due to pathologies in the small and medium airways (27). An infection process in the small airways could lead to the release of inflammatory mediators such as proteases and elastases (28). As consequences, the airway epithelium would be infiltrated by inflammatory cytokines and free radicals, promoting bronchial wall thickening and obstruction of small airways, as well as persistent bronchial dilatation (29). The above condition would lead to reduction in the effective cross-sectional area of the peripheral airways, thus increasing airway resistance, and impairing the lung elastic capacity accordingly. Guan et al. (24). showed that airway reversibility in bronchiectasis patients was associated with poorer lung function. Furthermore, a study on COPD patients showed that the reactance indices X5, Fres and the resistance Z5 and R5-R20 could help identify patients with FEV1% predicted <50% (30).

It is well known that chronic bronchial infection by *P. aeruginosa* was associated with a poorer prognosis than other pathogens, such as decline in lung function, higher severity score, more exacerbation, increased hospitalization, and greater mortality. Same results were found in this study. However, in our study, IOS parameters did not correlate with *P. aeruginosa* colonization. As we considered, it may be related to the low detection rate of *P. aeruginosa* (only 15% in the study). We noticed that a multicenter study conducted in Spain that focus on the impact of *P. aeruginosa* on lung function in bronchiectasis patients, which reported 25.7% of patients presented chronic bronchial infection (CBI) by *P. aeruginosa* (31). However, due to a different objective, the above study only included patients with CBI as their study population, and the detection rate of 25.7% was based on all patients with CBI. In our study, patients with negative or positive sputum culture were included. Because the subjects of the study were in stable condition, some patients had free of expectoration during the follow-up, resulting in a low positive rate of *P. aeruginosa* detection. This detection rate was comparable to that reported by Evans in the total bronchiectasis population (16/135) (32).

In the present study, all IOS parameters except for R20 and Rp all correlated negatively with lung spirometry variables and positively with airway trapping markers. Previous studies have demonstrated a good correlation between IOS parameters and spirometric parameters in diseases like asthma, COPD and cystic fibrosis (33, 34). In bronchiectasis, lower FEV1 is associated with increased bronchiectasis severity (35). Also, the extent of bronchiectasis and bronchial wall thickening is inversely associated with lung function (36, 37). Our results revealed that IOS parameters correlated well with both lung function and disease severity. This suggests that IOS may serve as an incredibly useful alternative to assess disease severity and monitor disease progression in bronchiectasis. However, unlike traditional spirometeric parameters, IOS was unable to distinguish patients with Pseudomonas aeruginosa infection from those without infections. IOS parameters also seem to be less influenced by previous history of hospitalization.

The present study has some limitations. First, it was a single-center investigation, and the findings could have been affected by selection bias. Second, although IOS is an emerging tool in the analysis of airway resistance and reactance in bronchiectasis, there are relatively few studies in this area, and no reference standard for IOS measurements has been established. Furthermore, future investigations cooperated by multi-center with a larger cohort of patients are required to address further questions such as the improvement rate of IOS measurement in the diagnostic test in patients with asthma, CODP, bronchiectasis, as well as in the overlap among these chronic airway diseases. We are looking forward to further study based on a large population that would corroborate our findings and establish standardized guidelines.

## Conclusion

In conclusion, this study supports the use of IOS to assess the severity of bronchiectasis. IOS parameters correlate well with disease severity stratified using multidimensional assessment tools that takes into account both clinical and radiological features. Furthermore, IOS parameters are shown to be effective in the prediction of airway reversibility in patients with bronchiectasis. In the future, IOS may function as an alternative to spirometry, to evaluate lung function in adults with bronchiectasis.

## Data Availability Statement

The raw data supporting the conclusions of this article will be made available by the authors, without undue reservation.

## Ethics Statement

The studies involving human participants were reviewed and approved by the Ethics Committee of the Fifth Affiliated Hospital of Sun Yat-Sen University (approve number [2015] K02-1). The patients/participants provided their written informed consent to participate in this study.

## Author Contributions

CTan participated in drafting the work, acquisition, analysis, and interpretation of data. DM participated in drafting the work, acquisition, analysis, and interpretation of data. CTu, MC, KW, XZ, YH, ZW, and JW participated in the data acquisition and interpretation. JH participated in the conception and design of the work. JL conceived of the study, and participated in its design and coordination and helped to draft the manuscript. All authors read and approved the final manuscript.

## Funding

JL was supported by the Zhuhai Science and Technology Innovation Bureau, Novel coronavirus epidemic prevention and control emergency research (Grant Numbers ZH22036302200021PWC) and the Guangdong Basic and Applied Basic Research Foundation (2020A1515011147). XZ received funding from the Fundamental Research Funds for the Central Universities (19ykpy51); the Medical Scientific Research Foundation of Guangdong Province of China (A2020176) and the Science and Technology Planning Project for medicine of Zhuhai City (ZH2202200012HJL). The authors state that neither the study design, results, interpretation of the findings, nor any other subject discussed in the submitted manuscript was dependent on financial support.

## Conflict of Interest

The authors declare that the research was conducted in the absence of any commercial or financial relationships that could be construed as a potential conflict of interest.

## Publisher's Note

All claims expressed in this article are solely those of the authors and do not necessarily represent those of their affiliated organizations, or those of the publisher, the editors and the reviewers. Any product that may be evaluated in this article, or claim that may be made by its manufacturer, is not guaranteed or endorsed by the publisher.

## Supplementary Material

Supplementary Figure 1 Flow chart of patient recruitment.

## Abbreviations

ABPA: Allergic bronchopulmonary aspergillosis
ATS: American Thoracic Society
AUC: Area under the curve
BDR: Bronchodilator response
BDT: Bronchial dilation test
BSI: Bronchiectasis Severity Index
COPD: Chronic obstructive pulmonary disease
ERS: European Respiratory Society
FDR: Frequency dependence of resistance
FEV1: Forced expiratory volume in one second
Fres: Resonant frequency
FVC: Forced vital capacity
GINA: Global Initiative for Asthma
HRCT: High-resolution computed tomography
IOS: Impulse oscillometry
IQR: Interquartile range
MEF75: Maximal expiratory flow at 75% of the FVC
MEF50: Maximal expiratory flow at 50% of the FVC
MEF25: Maximal expiratory flow at 25% of the FVC
MMEF: Maximal mid-expiratory flow
PCCM: Pulmonary and Critical Care Medicine
PEF: Peak expiratory flow
Rc: Central resistance
Rp: Peripheral resistance
RV: Residual volume
R5: Resistance at 5 Hz
R20: Resistance at 20 Hz
TLC: Total lung capacity
X5: Reactance at 5 Hz
Z5: Impedance value at 5 Hz.
