# Supplementary material for: The Role of Impulse Oscillometry in Evaluating Disease Severity and Predicting the Airway Reversibility in Patients With Bronchiectasis
Source: Front Med (Lausanne). 2022 Feb 25;9:796809. doi: 10.3389/fmed.2022.796809 (PMC9847491; doi:10.3389/fmed.2022.796809)
Supplement: Supplementary file 1 [file Table_1.DOCX]

**Supplementary Table 1. Comparison among bronchiectasis cohort in terms of FCAED parameter.**

| **Parameters** | **FACED stratification** | | | **p-value** | **a vs b** | **b vs c** | **a vs c** |
| --- | --- | --- | --- | --- | --- | --- | --- |
|  | **Mild (48)a** | **Moderate (18)b** | **Severe (8)c** |  |  |  |  |
| **Rc, kpa/l/s** | 0.3 (0.2, 0.3) | 0.3 (0.2, 0.3) | 0.3 (0.2, 0.3) | 0.98 | 0.94 | 0.98 | 0.80 |
| **Rp, kpa/l/s** | 0.3 (0.3, 0.4) | 0.7 (0.5, 1.0) | 0.9 (0.8, 1.0) | <0.001 | <0.001 | 0.11 | <0.001 |
| **Z5, kpa/l/s** | 0.5 (0.4, 0.6) | 0.7 (0.6, 0.8) | 0.8 (0.7, 1.0) | <0.001 | <0.001 | 0.45 | <0.001 |
| **R5, kpa/l/s** | 0.4 (0.3, 0.5) | 0.6 (0.6, 0.7) | 0.6 (0.6, 0.8) | <0.001 | <0.001 | 0.74 | 0.001 |
| **R20, kpa/l/s** | 0.3 (0.3, 0.4) | 0.4 (0.3, 0.4) | 0.3 (0.3, 0.4) | 0.24 | 0.096 | 0.34 | 0.84 |
| **R5-R20, kpa/l/s** | 0.1 (0.1, 0.2) | 0.3 (0.2, 0.4) | 0.3 (0.3, 0.4) | <0.001 | <0.001 | 0.45 | <0.001 |
| **X5, kpa/l/s** | -0.1 (-0.2, -0.1) | -0.4 (-0.6, -0.3) | -0.5 (-0.6, -0.4) | <0.001 | <0.001 | 0.16 | <0.001 |
| **Fres, Hz** | 17.3 (14.8, 21.8) | 28.3 (20.1, 33.0) | 26.9 (25.6, 33.1) | <0.001 | <0.001 | 0.58 | <0.001 |
| **FEV1, %pred** | 81.7 (69.5, 95.2) | 45.8 (29.8, 65.2) | 36.1 (28.0, 47.4) | <0.001 | <0.001 | 0.18 | <0.001 |
| **FVC, %pred** | 86.5 (81.0, 100.7) | 72.5 (63.6, 93.0) | 54.7 (37.2, 72.2) | <0.001 | 0.024 | 0.018 | <0.001 |
| **FEV1/FVC** | 74.8 (68.7, 79.0) | 46.8 (38.7, 63.1) | 49.4 (47.7, 55.2) | <0.001 | <0.001 | 0.60 | <0.001 |
| **RV, %pred** | 118.4 (96.2, 138.5) | 165.7 (119.0, 202.1) | 182.1 (127.8, 206.8) | <0.001 | 0.003 | 0.62 | 0.006 |
| **TLC, %pred** | 97.6 (87.2, 105.9) | 105.8 (92.2, 118.4) | 106.8 (93.8, 114.9) | 0.076 | 0.033 | 0.78 | 0.24 |
| **RV/TLC** | 44.6 (40.4, 49.0) | 61.2 (49.1, 72.1) | 72.0 (63.5, 78.9) | <0.001 | <0.001 | 0.030 | <0.001 |
| **MEF75, %pred** | 71.9 (51.1, 95.9) | 17.9 (9.4, 33.0) | 18.0 (12.1, 18.5) | <0.001 | <0.001 | 0.91 | <0.001 |
| **MEF50, %pred** | 47.9 (37.1, 68.1) | 14.6 (7.7, 20.8) | 11.1 (8.4, 17.3) | <0.001 | <0.001 | 0.58 | <0.001 |
| **MEF25, %pred** | 36.4 (24.6, 58.4) | 17.7 (11.2, 23.5) | 15.2 (13.0, 38.0) | <0.001 | <0.001 | 0.91 | 0.010 |
| **MMEF, %pred** | 45.3 (31.0, 65.0) | 15.2 (8.7, 22.0) | 12.3 (9.8, 21.5) | <0.001 | <0.001 | 0.87 | <0.001 |
| **PEF, %pred** | 87.9 (72.7, 103.7) | 40.2 (27.4, 59.0) | 34.0 (26.5, 40.3) | <0.001 | <0.001 | 0.29 | <0.001 |
| **VC IN, %pred** | 74.4 (60.7-88.3) | 60.6 (51.2-78.6) | 30.5 (27.2-50.1) | <0.001 | 0.10 | 0.009 | <0.001 |

Rc, central resistance; Rp, peripheral resistance; Z5, respiratory impedance at 5 Hz; R5 and R20, respiratory system resistance at 5 and 20Hz, respectively; X5, respiratory system reactance at 5Hz; Fres, resonant frequency; FEV1, forced expiratory volume in one second; FVC, forced vital capacity; RV, residual volume; TLC, total lung capacity; MEF, maximal expiratory flow; MMEF, maximal mid-expiratory flow; PEF, peak expiratory flow; VC IN, inspiratory vital capacity.
