# Supplementary material for: The Role of Impulse Oscillometry in Evaluating Disease Severity and Predicting the Airway Reversibility in Patients With Bronchiectasis
Source: Front Med (Lausanne). 2022 Feb 25;9:796809. doi: 10.3389/fmed.2022.796809 (PMC9847491; doi:10.3389/fmed.2022.796809)
Supplement: Supplementary file 2 [file Table_2.DOCX]

**Supplementary Table 2. Comparison among bronchiectasis cohort in terms of BSI parameter.**

| **Parameters** | **BSI stratification** | | | **p-value** | **a vs b** | **b vs c** | **a vs c** |
| --- | --- | --- | --- | --- | --- | --- | --- |
|  | **Mild (36)a** | **Moderate (27)b** | **Severe (11)c** |  |  |  |  |
| **Rc, kpa/l/s** | 0.3 (0.2, 0.3) | 0.3 (0.3, 0.3) | 0.2 (0.2, 0.3) | 0.25 | 0.68 | 0.13 | 0.13 |
| **Rp, kpa/l/s** | 0.3 (0.3, 0.5) | 0.5 (0.3, 0.7) | 0.8 (0.7, 1.0) | 0.001 | 0.021 | 0.037 | 0.001 |
| **Z5, kpa/l/s** | 0.5 (0.4, 0.5) | 0.6 (0.4, 0.8) | 0.8 (0.5, 1.0) | 0.002 | 0.015 | 0.069 | 0.003 |
| **R5, kpa/l/s** | 0.4 (0.3, 0.5) | 0.6 (0.4, 0.6) | 0.7 (0.4, 0.8) | 0.008 | 0.030 | 0.14 | 0.007 |
| **R20, kpa/l/s** | 0.3 (0.3, 0.4) | 0.3 (0.3, 0.4) | 0.4 (0.3, 0.4) | 0.71 | 0.50 | 0.70 | 0.55 |
| **R5-R20, kpa/l/s** | 0.1 (0.1, 0.2) | 0.2 (0.1, 0.3) | 0.3 (0.1, 0.4) | 0.002 | 0.012 | 0.049 | 0.003 |
| **X5, kpa/l/s** | -0.1 (-0.2, -0.1) | -0.3 (-0.4, -0.1) | -0.4 (-0.6, -0.3) | <0.001 | 0.004 | 0.055 | 0.002 |
| **Fres, Hz** | 16.4 (14.5, 20.4) | 23.6 (19.6, 28.1) | 26.1 (22.8, 34.9) | <0.001 | <0.001 | 0.17 | 0.001 |
| **FEV1, %pred** | 81.6 (19.8) | 64.2 (29.3) | 48.8 (26.2) | <0.001 | 0.007 | 0.14 | <0.001 |
| **FVC, %pred** | 91.5 (14.8) | 78.6 (20.7) | 65.4 (25.4) | <0.001 | 0.005 | 0.10 | <0.001 |
| **FEV1/FVC** | 71.7 (9.9) | 64.0 (19.1) | 58.5 (18.1) | 0.024 | 0.044 | 0.42 | 0.003 |
| **RV, %pred** | 118.4 (97.2, 138.0) | 140.3 (107.9, 196.1) | 138.2 (117.4, 180.0) | 0.037 | 0.018 | 0.83 | 0.092 |
| **TLC, %pred** | 97.9 (87.2, 108.3) | 101.8 (92.2, 114.6) | 96.0 (85.5, 109.1) | 0.44 | 0.25 | 0.34 | 0.75 |
| **RV/TLC** | 43.3 (40.6, 48.7) | 50.8 (45.7, 72.7) | 64.1 (53.9, 72.1) | <0.001 | <0.001 | 0.20 | <0.001 |
| **MEF75, %pred** | 74.0 (50.3, 102.1) | 42.6 (14.0, 71.1) | 18.5 (10.1, 37.7) | <0.001 | 0.005 | 0.16 | <0.001 |
| **MEF50, %pred** | 46.9 (39.0, 66.0) | 30.9 (12.0, 65.1) | 15.1 (8.3, 34.4) | 0.002 | 0.039 | 0.13 | <0.001 |
| **MEF25, %pred** | 33.8 (21.3, 53.3) | 25.0 (14.5, 61.6) | 19.1 (13.0, 53.5) | 0.23 | 0.24 | 0.43 | 0.13 |
| **MMEF, %pred** | 44.2 (30.1, 58.9) | 28.7 (12.3, 67.7) | 17.6 (8.3, 38.6) | 0.007 | 0.071 | 0.097 | 0.003 |
| **PEF, %pred** | 87.9 (72.3, 109.7) | 54.2 (36.9, 93.8) | 39.3 (27.4, 66.0) | <0.001 | <0.001 | 0.18 | <0.001 |
| **VC IN, %pred** | 78.3 (62.6-95.0) | 60.7 (47.4-77.2) | 58.8 (29.7-75.1) | 0.001 | 0.002 | 0.43 | 0.005 |

Rc, central resistance; Rp, peripheral resistance; Z5, respiratory impedance at 5 Hz; R5 and R20, respiratory system resistance at 5 and 20Hz, respectively; X5, respiratory system reactance at 5Hz; Fres, resonant frequency; FEV1, forced expiratory volume in one second; FVC, forced vital capacity; RV, residual volume; TLC, total lung capacity; MEF, maximal expiratory flow; MMEF, maximal mid-expiratory flow; PEF, peak expiratory flow; VC IN, inspiratory vital capacity.
