# Supplementary material for: The Role of Impulse Oscillometry in Evaluating Disease Severity and Predicting the Airway Reversibility in Patients With Bronchiectasis
Source: Front Med (Lausanne). 2022 Feb 25;9:796809. doi: 10.3389/fmed.2022.796809 (PMC9847491; doi:10.3389/fmed.2022.796809)
Supplement: Supplementary file 3 [file Table_3.DOCX]

**Supplementary Table 3. Comparison among bronchiectasis cohort in terms of Reiff parameter.**

| **Parameters** | **Reiff stratification** | | | **p-value** | **a vs b** | **b vs c** | **a vs c** |
| --- | --- | --- | --- | --- | --- | --- | --- |
|  | **Mild (47)a** | **Moderate (18)b** | **Severe (9)c** |  |  |  |  |
| **Rc, kpa/l/s** | 0.3 (0.2, 0.3) | 0.3 (0.3, 0.3) | 0.3 (0.3, 0.3) | 0.98 | 0.91 | 0.78 | 0.93 |
| **Rp, kpa/l/s** | 0.3 (0.3, 0.5) | 0.4 (0.3, 0.8) | 0.9 (0.8, 1.0) | <0.001 | 0.10 | 0.005 | <0.001 |
| **Z5, kpa/l/s** | 0.5 (0.4, 0.6) | 0.5 (0.4, 0.8) | 0.8 (0.7, 1.0) | 0.007 | 0.38 | 0.025 | 0.002 |
| **R5, kpa/l/s** | 0.5 (0.3, 0.6) | 0.5 (0.4, 0.7) | 0.6 (0.5, 0.8) | 0.042 | 0.54 | 0.060 | 0.013 |
| **R20, kpa/l/s** | 0.3 (0.3, 0.4) | 0.3 (0.3, 0.4) | 0.3 (0.3, 0.4) | 0.95 | 0.94 | 0.80 | 0.77 |
| **R5-R20, kpa/l/s** | 0.1 (0.1, 0.2) | 0.1 (0.1, 0.3) | 0.3 (0.3, 0.4) | 0.005 | 0.53 | 0.027 | <0.001 |
| **X5, kpa/l/s** | -0.1 (-0.3, -0.1) | -0.2 (-0.4, -0.1) | -0.5 (-0.6, -0.4) | <0.001 | 0.20 | 0.005 | <0.001 |
| **Fres, Hz** | 17.9 (14.9, 23.8) | 19.0 (15.7, 30.5) | 27.8 (25.6, 29.2) | 0.007 | 0.48 | 0.045 | 0.001 |
| **FEV1, %pred** | 75.7 (22.6) | 73.6 (31.7) | 36.3 (12.1) | <0.001 | 0.77 | 0.002 | <0.001 |
| **FVC, %pred** | 86.4 (17.3) | 88.0 (20.8) | 54.3 (16.8) | <0.001 | 0.75 | <0.001 | <0.001 |
| **FEV1/FVC** | 69.5 (13.1) | 66.0 (19.2) | 54.9 (17.1) | 0.036 | 0.40 | 0.16 | 0.005 |
| **RV, %pred** | 126.9 (96.5, 157.9) | 120.1 (103.1, 159.8) | 180.0 (125.4, 202.1) | 0.028 | 0.64 | 0.072 | 0.030 |
| **TLC, %pred** | 99.2 (87.4, 112.3) | 101.9 (88.9, 112.4) | 102.1 (91.7, 109.1) | 0.91 | 0.67 | 0.80 | 0.88 |
| **RV/TLC** | 46.6 (40.5, 58.2) | 48.3 (42.6, 54.1) | 65.8 (63.0, 77.3) | 0.001 | 0.54 | 0.002 | <0.001 |
| **MEF75, %pred** | 60.8 (37.3, 94.9) | 55.7 (17.6, 94.0) | 17.7 (11.8, 21.8) | 0.001 | 0.48 | 0.024 | <0.001 |
| **MEF50, %pred** | 46.0 (26.8, 69.0) | 44.7 (14.1, 55.9) | 9.3 (7.0, 19.5) | <0.001 | 0.28 | 0.010 | <0.001 |
| **MEF25, %pred** | 28.9 (22.5, 57.5) | 28.2 (13.6, 53.2) | 16.1 (13.0, 36.7) | 0.065 | 0.20 | 0.44 | 0.025 |
| **MMEF, %pred** | 40.5 (24.6, 65.3) | 36.8 (13.6, 54.2) | 11.0 (8.8, 21.4) | 0.002 | 0.26 | 0.027 | <0.001 |
| **PEF, %pred** | 84.0 (54.2, 100.4) | 75.8 (47.3, 93.8) | 32.0 (26.6, 41.2) | <0.001 | 0.45 | 0.002 | <0.001 |
| **VC IN, %pred** | 68.8 (50.8-85.3) | 76.6 (62.3-87.0) | 29.7 (25.9-60.1) | 0.001 | 0.34 | <0.001 | 0.001 |

Rc, central resistance; Rp, peripheral resistance; Z5, respiratory impedance at 5 Hz; R5 and R20, respiratory system resistance at 5 and 20Hz, respectively; X5, respiratory system reactance at 5Hz; Fres, resonant frequency; FEV1, forced expiratory volume in one second; FVC, forced vital capacity; RV, residual volume; TLC, total lung capacity; MEF, maximal expiratory flow; MMEF, maximal mid-expiratory flow; PEF, peak expiratory flow; VC IN, inspiratory vital capacity.
