# Supplementary material for: The Role of Impulse Oscillometry in Evaluating Disease Severity and Predicting the Airway Reversibility in Patients With Bronchiectasis
Source: Front Med (Lausanne). 2022 Feb 25;9:796809. doi: 10.3389/fmed.2022.796809 (PMC9847491; doi:10.3389/fmed.2022.796809)
Supplement: Supplementary file 4 [file Table_4.DOCX]

**Supplementary Table 4. Comparison among bronchiectasis cohort in terms of Bhalla parameter.**

| **Parameters** | **Bhalla stratification** | | | **p-value** |  |  |  |
| --- | --- | --- | --- | --- | --- | --- | --- |
|  | **Mild (49)a** | **Moderate (23)b** | **Severe (2)c** |  | **a vs b** | **b vs c** | **a vs c** |
| **Rc, kpa/l/s** | 0.3 (0.2, 0.3) | 0.3 (0.2, 0.3) | 0.3 (0.3, 0.3) | 0.34 | 0.16 | 0.76 | 0.59 |
| **Rp, kpa/l/s** | 0.3 (0.3, 0.6) | 0.4 (0.3, 1.0) | 0.7 (0.7, 0.8) | 0.080 | 0.090 | 0.80 | 0.058 |
| **Z5, kpa/l/s** | 0.5 (0.4, 0.6) | 0.6 (0.4, 0.8) | 0.6 (0.5, 0.7) | 0.32 | 0.15 | 0.92 | 0.51 |
| **R5, kpa/l/s** | 0.5 (0.4, 0.6) | 0.5 (0.4, 0.7) | 0.5 (0.4, 0.6) | 0.56 | 0.28 | 0.76 | 0.87 |
| **R20, kpa/l/s** | 0.3 (0.3, 0.4) | 0.3 (0.3, 0.3) | 0.3 (0.3, 0.3) | 0.081 | 0.12 | 0.27 | 0.072 |
| **R5-R20, kpa/l/s** | 0.1 (0.1, 0.2) | 0.2 (0.1, 0.3) | 0.2 (0.1, 0.3) | 0.080 | 0.036 | 0.84 | 0.30 |
| **X5, kpa/l/s** | -0.2 (-0.3, -0.1) | -0.2 (-0.6, -0.1) | -0.4 (-0.4, -0.3) | 0.073 | 0.070 | 0.88 | 0.072 |
| **Fres, Hz** | 19.2 (15.2, 24.8) | 22.7 (17.0, 30.6) | 24.6 (23.2, 26.1) | 0.13 | 0.081 | 0.69 | 0.21 |
| **FEV1, %pred** | 77.1 (24.4) | 58.1 (28.5) | 45.9 (12.7) | 0.007 | 0.005 | 0.56 | 0.080 |
| **FVC, %pred** | 87.3 (18.0) | 76.3 (22.9) | 52.1 (29.8) | 0.010 | 0.030 | 0.17 | 0.010 |
| **FEV1/FVC** | 70.0 (14.2) | 59.4 (16.2) | 75.5 (28.8) | 0.020 | 0.006 | 0.21 | 0.61 |
| **RV, %pred** | 121.3 (98.5, 163.5) | 138.8 (107.9, 180.0) | 109.2 (101.0, 117.4) | 0.21 | 0.13 | 0.16 | 0.53 |
| **TLC, %pred** | 99.3 (89.0, 112.6) | 101.6 (87.1, 112.3) | 73.1 (54.5, 91.7) | 0.21 | 0.92 | 0.089 | 0.081 |
| **RV/TLC** | 46.9 (41.3, 59.8) | 49.1 (42.6, 65.8) | 63.5 (63.0, 64.1) | 0.23 | 0.38 | 0.27 | 0.11 |
| **MEF75, %pred** | 67.0 (37.3, 94.9) | 37.7 (11.8, 63.2) | 25.6 (18.3, 32.8) | 0.012 | 0.007 | 0.76 | 0.099 |
| **MEF50, %pred** | 46.2 (26.8, 69.0) | 20.9 (9.0, 46.0) | 26.9 (19.5, 34.4) | 0.015 | 0.005 | 0.92 | 0.26 |
| **MEF25, %pred** | 29.6 (20.9, 59.3) | 19.7 (13.2, 37.1) | 54.3 (53.5, 55.1) | 0.013 | 0.009 | 0.060 | 0.31 |
| **MMEF, %pred** | 42.4 (24.6, 65.3) | 21.4 (9.5, 44.1) | 32.0 (25.4, 38.6) | 0.017 | 0.005 | 0.62 | 0.56 |
| **PEF, %pred** | 77.9 (54.2, 100.6) | 59.0 (27.5, 85.9) | 36.6 (32.0, 41.2) | 0.019 | 0.023 | 0.37 | 0.052 |
| **VC IN, %pred** | 70.8 (54.0-86.8) | 61.3 (51.2-78.1) | 44.3 (29.7-58.8) | 0.16 | 0.24 | 0.23 | 0.10 |

Rc, central resistance; Rp, peripheral resistance; Z5, respiratory impedance at 5 Hz; R5 and R20, respiratory system resistance at 5 and 20Hz, respectively; X5, respiratory system reactance at 5Hz; Fres, resonant frequency; FEV1, forced expiratory volume in one second; FVC, forced vital capacity; RV, residual volume; TLC, total lung capacity; MEF, maximal expiratory flow; MMEF, maximal mid-expiratory flow; PEF, peak expiratory flow; VC IN, inspiratory vital capacity.
