# Supplementary material for: The Role of Impulse Oscillometry in Evaluating Disease Severity and Predicting the Airway Reversibility in Patients With Bronchiectasis
Source: Front Med (Lausanne). 2022 Feb 25;9:796809. doi: 10.3389/fmed.2022.796809 (PMC9847491; doi:10.3389/fmed.2022.796809)
Supplement: Supplementary file 5 [file Table_5.DOCX]

**Supplementary Table 5. Comparison among bronchiectasis cohort in terms of BRICS parameter.**

| **Parameters** | **BRICS stratification** | | | **p-value** |  |  |  |
| --- | --- | --- | --- | --- | --- | --- | --- |
|  | **Mild (25)a** | **Moderate (39)b** | **Severe (10)c** |  | **a vs b** | **b vs c** | **a vs c** |
| **Rc, kpa/l/s** | 0.3 (0.3, 0.3) | 0.3 (0.2, 0.3) | 0.3 (0.2, 0.3) | 0.36 | 0.35 | 0.35 | 0.23 |
| **Rp, kpa/l/s** | 0.4 (0.3, 0.6) | 0.3 (0.3, 0.5) | 0.8 (0.6, 1.0) | 0.045 | 0.61 | 0.022 | 0.022 |
| **Z5, kpa/l/s** | 0.5 (0.4, 0.7) | 0.5 (0.4, 0.7) | 0.7 (0.5, 0.9) | 0.26 | 0.59 | 0.12 | 0.19 |
| **R5, kpa/l/s** | 0.5 (0.4, 0.6) | 0.5 (0.3, 0.6) | 0.6 (0.4, 0.8) | 0.49 | 0.50 | 0.26 | 0.52 |
| **R20, kpa/l/s** | 0.4 (0.3, 0.4) | 0.3 (0.3, 0.4) | 0.3 (0.3, 0.3) | 0.14 | 0.20 | 0.21 | 0.079 |
| **R5-R20, kpa/l/s** | 0.2 (0.1, 0.2) | 0.1 (0.1, 0.3) | 0.3 (0.1, 0.3) | 0.20 | 0.69 | 0.094 | 0.10 |
| **X5, kpa/l/s** | -0.2 (-0.3, -0.1) | -0.2 (-0.3, -0.1) | -0.4 (-0.6, -0.3) | 0.040 | 0.70 | 0.020 | 0.016 |
| **Fres, Hz** | 19.3 (14.3, 25.0) | 18.3 (15.7, 24.8) | 25.6 (22.8, 29.2) | 0.29 | 0.77 | 0.14 | 0.17 |
| **FEV1, %pred** | 77.7 (20.1) | 70.9 (28.5) | 50.0 (28.7) | 0.021 | 0.30 | 0.045 | 0.003 |
| **FVC, %pred** | 88.3 (13.2) | 83.5 (23.7) | 67.2 (18.6) | 0.023 | 0.36 | 0.050 | <0.001 |
| **FEV1/FVC** | 70.1 (12.1) | 66.9 (14.9) | 58.8 (24.0) | 0.17 | 0.38 | 0.19 | 0.077 |
| **RV, %pred** | 132.8 (103.1, 165.3) | 122.2 (98.0, 150.6) | 153.4 (117.4, 206.4) | 0.15 | 0.35 | 0.056 | 0.24 |
| **TLC, %pred** | 105.7 (89.0, 113.2) | 97.6 (85.5, 104.8) | 103.1 (93.3, 121.6) | 0.17 | 0.082 | 0.25 | 0.94 |
| **RV/TLC** | 48.4 (44.0, 60.5) | 45.7 (41.0, 54.1) | 63.5 (53.9, 70.2) | 0.030 | 0.29 | 0.015 | 0.034 |
| **MEF75, %pred** | 71.1 (37.3, 91.2) | 56.3 (20.1, 88.1) | 16.2 (9.4, 37.7) | 0.045 | 0.40 | 0.037 | 0.018 |
| **MEF50, %pred** | 47.5 (26.8, 63.6) | 41.4 (17.6, 61.0) | 14.4 (6.9, 34.4) | 0.070 | 0.31 | 0.070 | 0.031 |
| **MEF25, %pred** | 28.9 (20.9, 57.6) | 25.9 (17.7, 50.4) | 14.5 (13.0, 55.1) | 0.46 | 0.36 | 0.54 | 0.28 |
| **MMEF, %pred** | 44.7 (24.6, 62.9) | 33.6 (18.4, 56.2) | 14.4 (7.3, 38.6) | 0.087 | 0.26 | 0.078 | 0.053 |
| **PEF, %pred** | 74.4 (63.0, 100.0) | 78.1 (47.0, 100.0) | 38.6 (32.0, 66.0) | 0.080 | 0.70 | 0.066 | 0.018 |
| **VC IN, %pred** | 73.7 (50.8-79.4) | 67.2 (57.2-89.1) | 59.7 (29.7-69.5) | 0.11 | 0.92 | 0.054 | 0.045 |

Rc, central resistance; Rp, peripheral resistance; Z5, respiratory impedance at 5 Hz; R5 and R20, respiratory system resistance at 5 and 20Hz, respectively; X5, respiratory system reactance at 5Hz; Fres, resonant frequency; FEV1, forced expiratory volume in one second; FVC, forced vital capacity; RV, residual volume; TLC, total lung capacity; MEF, maximal expiratory flow; MMEF, maximal mid-expiratory flow; PEF, peak expiratory flow; VC IN, inspiratory vital capacity.
