# Supplementary material for: The Role of Impulse Oscillometry in Evaluating Disease Severity and Predicting the Airway Reversibility in Patients With Bronchiectasis
Source: Front Med (Lausanne). 2022 Feb 25;9:796809. doi: 10.3389/fmed.2022.796809 (PMC9847491; doi:10.3389/fmed.2022.796809)
Supplement: Supplementary file 6 [file Table_6.DOCX]

**Supplementary Table 6. Clinical characteristics of patients with bronchiectasis presenting with either positive or negative bronchodilatation test (BDT).**

|  | **Total (n=74)** | **BDT negative**  **(n=53)** | **BDT positive**  **(n=21)** | **p-value** |
| --- | --- | --- | --- | --- |
| Age(yrs) | 60.4 (11.2) | 60.1 (10.3) | 61.2 (13.5) | 0.71 |
| Gender |  |  |  | 0.74 |
| Male | 41 (55%) | 30 (57%) | 11 (52%) |  |
| Female | 33 (45%) | 23 (43%) | 10 (48%) |  |
| Height (cm) | 161.2 (7.9) | 161.6 (9.0) | 160.0 (4.3) | 0.45 |
| Weight (kg) | 58.8 (12.7) | 58.7 (13.1) | 58.9 (12.0) | 0.95 |
| BMI (kg·m^–2^) | 22.6 (4.6) | 22.4 (4.2) | 23.2 (5.5) | 0.54 |
| Smoking |  |  |  | 0.79 |
| Non-smoker | 44 (59%) | 31 (58%) | 13 (62%) |  |
| Active smoker | 30 (41%) | 22 (42%) | 8 (38%) |  |
| Disease years | 3.0 (1.0-6.0) | 3.0 (1.0-6.0) | 3.0 (2.0-10.0) | 0.23 |
| Exacerbation | 0.0 (0.0-2.0) | 0.0 (0.0-2.0) | 1.0 (0.0-1.0) | 0.47 |
| Hospitalization |  |  |  | 0.26 |
| Yes | 64 (86%) | 44 (83%) | 20 (95%) |  |
| No | 10 (14%) | 9 (17%) | 1 ( 5%) |  |
| mMRC score |  |  |  | 0.25 |
| 0 | 2 ( 3%) | 2 ( 4%) | 0 ( 0%) |  |
| 1 | 33 (45%) | 26 (49%) | 7 (33%) |  |
| 2 | 25 (34%) | 18 (34%) | 7 (33%) |  |
| 3 | 11 (15%) | 5 ( 9%) | 6 (29%) |  |
| 4 | 3 ( 4%) | 2 ( 4%) | 1 ( 5%) |  |
| BSI scores | 5.0 (3.0-8.0) | 4.0 (2.0-7.0) | 6.0 (4.0-8.0) | 0.077 |
| BSI stratification |  |  |  | 0.18 |
| Mild | 36 (49%) | 29 (55%) | 7 (33%) |  |
| Moderate | 27 (36%) | 16 (30%) | 11 (52%) |  |
| Severe | 11 (15%) | 8 (15%) | 3 (14%) |  |
| FACED | 1.0 (1.0-3.0) | 1.0 (0.0-2.0) | 3.0 (2.0-4.0) | <0.001 |
| FACED stratification |  |  |  | 0.001 |
| Mild | 48 (65%) | 41 (77%) | 7 (33%) |  |
| Moderate | 18 (24%) | 8 (15%) | 10 (48%) |  |
| Severe | 8 (11%) | 4 ( 8%) | 4 (19%) |  |
| Lobes affected | 4.0 (2.0-5.0) | 3.0 (2.0-5.0) | 5.0 (3.0-6.0) | 0.011 |

Categorical data expressed as n (%), continuous data expressed either with mean (SD) or median (IQR) as appropriate. mMRC, modified Medical Research Council.
